# The Advantage of Using Video Laryngoscope in Puncture and Incisional Drainage of Peritonsillar Abscess: A Case Report

**DOI:** 10.21980/J8G935

**Published:** 2025-01-31

**Authors:** Daisuke Goto, Jin Takahashi, Hiraku Funakoshi

**Affiliations:** * Tokyo Bay Urayasu Ichikawa Medical Center, Department of Emergency and Critical Care Medicine, Urayasu, Japan

## Abstract

This case report demonstrates the use of a video laryngoscope to aid in the incision and drainage of a peritonsillar abscess in a 30-year-old male. This technique, which has not been widely discussed in the literature, provided enhanced visualization, overcoming challenges like poor access due to trismus and poor lighting. The video laryngoscope improved safety, offered clearer guidance, and provided valuable teaching opportunities, particularly for less experienced physicians and residents. This case contributes to the literature by illustrating how the video laryngoscope can function as both a therapeutic tool and an educational resource, improving the management of peritonsillar abscesses and potentially reducing complications.

**Topics:**

Peritonsillar abscess, peritonsillar aspiration, peritonsillar incision, video laryngoscope.

[Fig f1-jetem-10-1-v22]

**Figure f1-jetem-10-1-v22:**
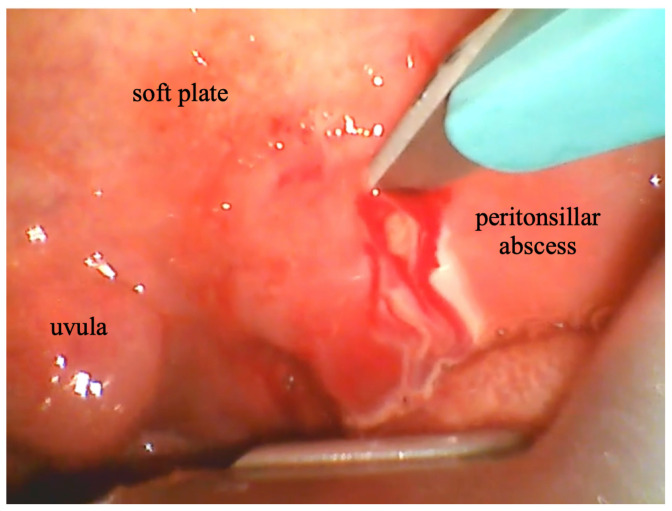
Video Link: https://youtu.be/FUiIserPgk0

## Brief introduction

Peritonsillar abscess is one of the most common ENT diseases presenting in the emergency department with an incidence of approximately 1 in 10,000.^1^ It is more common among the adolescent population, although it can occur in any group.^2^ Drainage by aspiration or incision is an effective treatment option for rapid disease cure and symptom relief.^3^ However, inexperienced physicians may encounter difficulties in the drainage procedure, such as poor access due to trismus and poor lighting. To counter these and other difficulties, previous studies recommended the use of some tools as a therapeutic adjunct.^4^ This time we propose the use of a video laryngoscope as the solution which has never been considered in the literature.

## Presenting concerns and clinical findings

A 30-year-old male with no past medical history presented to the emergency department with a seven-day history of fever, sore throat, and trismus. He had not been taking any medication. The general appearance was good. His face didn’t have facial swelling and change in phonation. Intraoral examination revealed erythema and swelling of the left palatine tonsil with mild palatine deviation. There was trismus with the space between the incisors being two finger widths. The laboratory results showed that the white blood cell count was 10,300 /μL and CRP level was 1.24 mg/dL. Contrast-enhanced computed tomography confirmed the presence of a left peritonsillar abscess (12mm × 15mm × 14mm). We diagnosed a peritonsillar abscess based on physical and imaging findings.

## Significant findings

Incision of the peritonsillar abscess was performed with the assistance of the C-MAC video laryngoscope which provided a clear, illuminated, and unobstructed view of the incision site. Local anesthesia with 1% xylocaine was administered, and the abscess was incised with a scalpel and drained with a forceps. (Supplementary video)

## Patient course

Immediately after the incision, the patient’s sore throat improved. He was followed up in the clinic without additional aspiration or incision and treated with antimicrobial therapy (ceftriaxone 2g daily intravenously for first four days and amoxicillin-clavulanate three times daily orally for next seven days). His symptoms and intraoral clinical findings completely improved without any complications in a week.

## Discussion

Peritonsillar abscess is a common condition encountered in the emergency department.^1^ Drainage by aspiration or incision is an effective treatment option for rapid disease cure and symptom relief.^2^ However, inexperienced physicians may encounter difficulties, such as poor access due to trismus and poor lighting, in the drainage procedure. These factors limit the visual field, making it difficult for the attending physician to observe and teach with the same visual field.^1^ Therefore, this situation makes it more difficult for residents to acquire precise procedural skills. A video laryngoscope can address these challenges and be a valuable training tool. Furthermore, the video laryngoscope has several advantages; the light from the laryngoscope facilitates observation of the posterior pharynx, the blade handle is positioned above the patient’s tongue, which prevents obstruction of the physician’s view, and the weight of the blade overcomes the obstacle of opening the mouth. The video laryngoscope doesn’t make it harder to reach the tonsil because the curved blade follows the tongue and the handle is below the patient’s mouth without obscuring the view holding by hand. The difference in method from intubation is the shallower blade insertion and the way the blade is held. These strengths provide enhanced visualization of the posterior pharynx and facilitate exposure of the affected area. Neither aspiration nor incision of a peritonsillar abscess carries a significantly higher risk of carotid artery injury as long as attention is paid to the depth and angle of the incision during drainage of the abscess.^5^ Using the video laryngoscope might help visualize the abscess, potentially reducing the risk of complications for both aspiration and incision.

Moreover, the attending physician can monitor and guide the residents through the procedure using the video screen, which enhances the educational aspect. The disadvantage is that the field of view is inverted, upside down from that under direct vision. Because the video screen of the C-MAC video laryngoscope can be addressed by two rotation axes, the disadvantage could be overcome if the operator or attending physician needs it to be.^6^ Also, local anesthesia may be useful to prevent the patient’s vomiting reflex when inserting the video. In this case, a video laryngoscope aided in securing a clear field of view and provided real-time guidance during the incision of a peritonsillar abscess. In conclusion, the video laryngoscope could be an effective training tool and therapeutic adjunct for managing peritonsillar abscess, and it makes it possible to perform the procedure safely.

## Supplementary Information


